# First Documented Uses of Caves along the Coast of Albania by Mediterranean Monk Seals (*Monachus monachus*, Hermann 1779): Ecological and Conservation Inferences

**DOI:** 10.3390/ani12192620

**Published:** 2022-09-29

**Authors:** Luigi Bundone, Gema Hernandez-Milian, Nexhip Hysolakoj, Rigers Bakiu, Tatjana Mehillaj, Lorela Lazaj, Hua Deng, Amy Lusher, Giulio Pojana

**Affiliations:** 1 Archipelagos-Ambiente e Sviluppo, Italia, Calle Asiago n° 4, 30132 Venezia, Italy; 2 Department of Philosophy and Cultural Heritage, Ca’ Foscari University of Venice, Dorsoduro 3484/d, 30123 Venezia, Italy; 3 Centre Oceanographic Vigo-Spanish National Research Council (COV-CSIC), Subida Radio Faro 50, 36390 Vigo, Spain; 4 Regional Administration of Protected Areas Vlorë (RAPA Vlorë), Rruga Sazani, Plazhi i Vjetër, 9401 Vlorë, Albania; 5 Albanian Center for Environmental Protection and Sustainable Development (ACEPSD), Rruga Aleksandër Moisiu Pallati 53/1, Kati i Pare, 1000 Tirana, Albania; 6 Norwegian Institute for Water Research (NIVA), Økernveien 94, 0579 Oslo, Norway

**Keywords:** Mediterranean monk seal, Adriatic Sea, habitat, diet, microplastics

## Abstract

**Simple Summary:**

Present and past information on endangered species distribution, biology and ecology is essential to implement conservation measures. The first documented use of habitat by the Mediterranean monk seal along the coasts of Albania is here presented. This was confirmed through the use of infrared camera traps and by the findings of a seals’ scat, proving the use of two caves in the study area. The results of the analysis of the scat revealed the last meal (three fish species) as well as microplastics and other non-food items. The relevance of this research goes beyond national borders, providing essential upgraded information for the species in Albania, and it also represents important data on an Adriatic–Ionian Basin context for the protection and recovery of the species.

**Abstract:**

Information on the habitat use of the Mediterranean monk seal (*Monachus monachus*) along the coast of Albania (Adriatic and Ionian Sea) has so far been limited to vague and generalised data. A survey conducted in the National Marine Park Karaburun-Sazan in the summer of 2019 identified two marine caves with morphological characteristics best suited for use by such species. The two caves were subsequently equipped with infrared camera traps in 2020. The recovery of a scat in one of the caves during the 2019 survey and the photographic material obtained confirmed the use of the cave. This research provides the first documentation of marine cave habitat use by the Mediterranean monk seal in Albania. Quantitative and qualitative assessment of specimens frequenting the area could not be performed due to the limited data obtained on seal presence along the Albanian coasts. Nevertheless, the retrieved information is relevant for Albania and for the species conservation. The collected scat was analysed for trophic and anthropogenic contamination data. Three species (gilthead sea bream, European sea bass, and garfish), as well as four anthropogenic items (including a piece of nylon net), were identified. The inferences resulting from the analyses of the data presented in this study provided additional information on the ecology of the species and its conservation priorities, which need to be contextualized at the Adriatic–Ionian regional scale.

## 1. Introduction

The Mediterranean monk seal (*Monachus monachus*, Hermann 1779) is classified by the International Union for the Conservation of Nature (IUCN) as an endangered species throughout its known distribution, and, along the Albanian coasts, it has been reported as possibly extinct [1]. The species’ main known reproductive groups have been found in Cabo Blanco (Mauritania and Western Sahara) and Madeira (Portugal) in the Atlantic Ocean, as well as in the Mediterranean Sea, along the Eastern Basin coasts (Greece and Turkey). The population inhabiting the Mediterranean Sea is estimated to consist of about 350–450 animals [2,3]. Occasional monk seal sightings have been reported outside of the above-mentioned Mediterranean nuclei. Sightings have been increasing in frequency particularly in the Adriatic–Ionian Basin and Levantine and Tyrrhenian Seas [4,5]; however, an exhaustive assessment of the species in these areas was lacking.

The historical presence of monk seal in Albania has been previously acknowledged by the UNEP-MAP (United Nations Environment Programme—Mediterranean Action Plan), GFCM (General Fisheries Commission for the Mediterranean), SPA-RAC (Specially Protected Areas Regional Activity Centre), and IUCN. Although these accredited bodies have documented the species’ potential status along the Albanian coast, no specific information is currently available at the population or individual level, or on the specificity of the used habitat by these animals.

Pinnipeds (seals, sea lions, and walruses) are marine mammals that preserved a connection to the mainland [6], where they spend time to haul out and give birth during the reproductive period. The use of coastal, dry land territories exposed these species to anthropogenic impacts such as direct human disturbance and noise [7]. The Mediterranean monk seal progressively moved to occupy areas less accessible to mankind due to anthropic pressures, including direct and indirect killings, and the habitat loss. The species mainly uses marine caves along cliff-bound coastal areas to haul out, rest, and give birth, at least over the past centuries [2,8]. These locations are usually difficult for humans to access; moreover, in such environments, the monitoring and collection of samples, such as scats or carcasses, becomes a challenging task. Bibliographical investigation of monk seal sightings and presence, including grey literature, is essential for understanding the relevance of a specific area for the species conservation. A recent study [9] compiled this information for the Albanian coast as a general framework for such a purpose. Despite the intensive bibliography search, the information retrieved did not provide any data with references to the specific habitat used by monk seals along this coastline.

The trophic ecology of the Mediterranean monk seal is currently scarce and mostly based on stomach contents from stranded animals, e.g., [10,11,12,13]. However, there are several non-systematic reports describing insights on the diet of this species. The knowledge of the dietary preferences of the species provides information on their habitat use (coastal area, platform, slope, or oceanic pelagic ecosystems) and targeted prey species. This becomes essential to assess the anthropogenic pressures that seals might be exposed to at sea. Dietary studies are carried out along Northern European coasts using scats collected at seal haul-outs rather than from carcasses, which are more difficult to recover. Only few carcasses regularly strand and appear in accessible areas; when they are found, they usually are in an advanced decomposition state. However, seal scats are usually collected from their haul-out and resting areas once animals leave (e.g., [14]). Taking into account the fact that the Mediterranean monk seal population is smaller than those of other pinniped populations from Northern Europe, the use of scats represents a practical and efficient tool to obtain information on the feeding ecology. 

Along with human disturbance, marine litter has become the focus of many investigations. This is especially the case for risk of ingestion and impact towards marine biota, including the potential effects throughout the food web. Most of the previous studies that aimed to assess the incidence of marine litter have been carried out analysing the digestive tracts of animals following the protocol for top predators available in the literature, which have been modified from dietary investigations [15]. The use of carcasses of marine mammals is a challenging task because only a small proportion of animals strands in good conditions to recover the samples. Although macrolitter ingestion is regularly reported, microlitter ingestion needs to be assessed under specific lab conditions and material [15]. In addition, seal carcasses occur in lower numbers than other marine mammals (Gema Hernandez-Milian, *pers.comm.*), so the use of scats for these studies is an alternative tool for the study of these pollutants, e.g., [14].

Albania has been a candidate for the accession to the European Union (EU) since 2014, with negotiations beginning in 2020. The EU agenda includes the protection of each country’s marine environment in connection with other European countries through the Marine Strategy Framework Directive (MSFD). The implementation of the MSFD requires the knowledge of ecosystems and the ecology, behaviour, and interactions with human activities of different species. To the best of our knowledge, no information is available on the monk seal feeding ecology in Albania, including grey literature. Dietary findings are important to understand the role of this top predator in the Mediterranean Sea. Data obtained from these studies are considered relevant within the Marine Strategy Framework Directive (Directive 2008/56/EC), which aims to achieve a good environmental status of European waters. In particular, Descriptor 4 (D4), focused on food webs, needs to be supported with information on the feeding ecology of all compartments of the marine ecosystems, in particular of top predators, to obtain valid indicators.

The aim of this study was to present the first testimony of effective and specific habitat used by the monk seal along the Albanian coast and to contribute to the abundance and distribution of this species in the Mediterranean Sea. In addition, it aimed to provide a better understanding of the ecological aspects and interactions with human activities of a yet poorly known species through insights on their diet and incidence of microplastics.

## 2. Materials and Methods

### 2.1. Study Area Description

The coast of Albania is characterized by low coastal formations along the central and northern part of its sea borders (Southern Adriatic Sea) and erosive coastline from Vlorë to Sarandë (Northern Ionian Sea) along the southern parts of the country. The southern coast is predominantly categorized by cliff coasts (from Karaburun-Sazan to Sarandë) interrupted in some sectors by pebble and sandy beaches. It lowers down towards Butrinti Bay, close to the southern border, into an accumulative coastline. The Peninsula of Karaburun and the island of Sazan are the areas where marine caves are mostly concentrated [16,17,18]. The territory was established as a protected area in 2010, including the correspondent aquatic territory (Marine National Park “Karaburun-Sazan”, hereafter MNP K-S).

### 2.2. Identification of Caves and Seals

**Figure 1:**
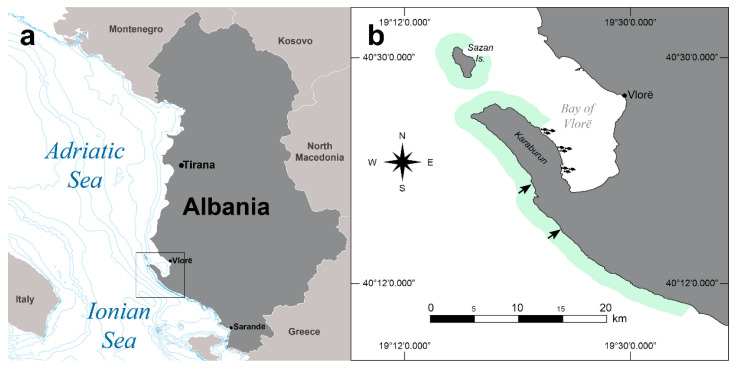
(**a**) Map of Albania highlighting the study area enlarged in (**b**). (**b**) Territory of the Marine National Park Karaburun-Sazan. In the map, the rough location of the 2 caves (arrows) equipped with infrared cameras to monitor Mediterranean monk seals’ effective use along the western coast of the park is represented. The position of the fish farms in the area (fish-shaped icons) is located on the eastern coast of the Karaburun Peninsula (Bay of Vlorë). The exact geographical position of the caves is not reported to protect the locations.

In August 2019, a survey aimed to investigate suitable and potential habitats available for the Mediterranean monk seal was carried out along the ~65 km long coastline of the MNP K-S (Figure 1a,b). All caves along the coast were investigated, and the ones suitable for monk seal use were mapped and described [9]. In addition, specific anthropogenic uses in the area of the study were also recorded (e.g., aquaculture sea cage farms).

Among the surveyed and mapped caves, the ones showing the best characteristics for the use by the investigated species were equipped with infrared camera traps (Guard 1, Keep Guard) set on a motion detection mode. The cameras were exchanged with a variable frequency to verify monk seal presence while keeping to the minimum any possible environmental disturbance to the specimens. These procedures were carried out according to the weather conditions and the COVID-19 global pandemic.

Cameras were installed in the caves on 28 January 2020 and retrieved on 24 May 2020 for summer, reinstalled on 10 October, and finally retrieved on 21 April 2021. Additionally, the cameras were checked 6 times within the overall period to verify their functioning. On two occasions, the cameras in cave 1 were found broken down, and the photos collected in the periods 10 October 2020 to 16 December 2020 and 22 February 2021 to 04 March 2021 were lost. Over the total period, 1952 pictures were recorded by the cameras.

It should be noted that, during the summer months, the cameras were designed to be retrieved in order to avoid being stolen or destroyed. These actions were carried out due to the presence of tourists in the area and some potential illegal activities (e.g., smuggling) within the park territory, as well as due to the difficulties in efficient patrol, control, and regulation of the area. Cameras were planned to be reinstalled in the caves during the autumn months. All collected pictures were analysed following the characteristics of the fur and presence of scars for photo-identification.

During 2019 and 2020, opportunistic pictures were taken by the general public within a citizen science activity promoted by the managing authority of the MNP K-S, the Regional Administration of Protected Areas Vlorë (RAPA Vlorë). These pictures were previously analysed for the identification of seals and published in [9] and were compared with the pictures obtained from the infrared cameras within the present study.

### 2.3. Identification of Prey Remains and Microplastics

**Figure 2:**
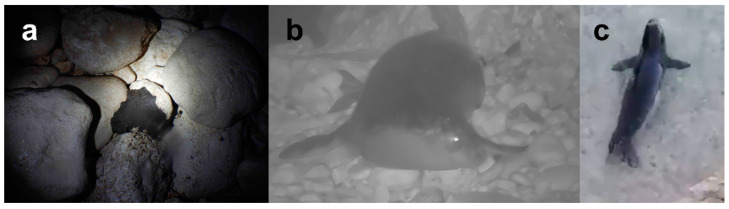
(**a**) Mediterranean scat collected in cave 1; (**b**) picture of the monk seal specimen caught on infrared camera in cave 1; (**c**) picture extracted from a video of monk seal collected in 2019 by RAPA Vlorë within the cited citizen science project.

A monk seal scat was recovered from one of the mapped caves (Figure 2a). The scat was collected in a single-use plastic bag (low-density polyethylene, LDPE). It was sent to the Marine Research Institute (IIM-CSIC), Vigo (Spain), and stored in a freezer for further analysis. Information about the conditions of the cave, presence of debris and other animals, as well as the type of clothes used during the research within the cave was recorded. The scat was processed for both prey identification and incidence of anthropogenic debris, in particular to investigate the potential trophic transfer of these pollutants. The scat analysis is a good technique for the description of prey items and detecting the incidence of microplastics, e.g., [10,14,15], ingested through their prey in a small population of seals, when carcasses are unlikely to be recovered.

A review of the feeding ecology of the species was carried out including both scientific publications and grey literature. This review was compiled and collected by “Archipelagos-ambiente e sviluppo, Italia” members since 1997 and using Scopus, ISI Web of Science, and Google Scholar. All collected information is summarized in the Results section.

The laboratory was previously prepared to carry out both diet and marine debris (microplastic and anthropogenic materials, >150 µm) analysis following the protocol published for marine vertebrates [15]. All working surfaces and the sink were cleaned the day before with ethanol 70% and paper; the laboratory was closed overnight to allow any suspended microplastics to settle to the ground. A single researcher was allowed to work on the scat analysis to avoid airborne contamination. Three blanks were located around the working area to test the airborne contamination, consisting of three GF/C microfiber filter papers; one of them was moistened with pre-filtered water. They were inspected in the same way as filtered samples for potential microplastic airborne contamination. The sample was soaked in pre-filtered water overnight for a better separation. On the following day, it was washed through a set of sieves (1000, 350, and 120 μm) with pre-filtered water. Large items (diet, parasites, and large anthropogenic items) were recovered from the first two sieves. The remains collected by the bottom sieve were transferred to a glass jar, previously cleaned with pre-filtered water, and stored for further microplastics analysis. The sample was filtered under vacuum using a Buchner filter. The particles found were photographed, some of them measured, transferred to dry paper under an optical microscope (LEICA EZ4), and later sent to the NIVA microplastics laboratory for polymer identification using Fourier transform infrared spectroscopy (FTIR).

All samples were visually inspected under a stereomicroscope (Nikon SMZ745T, 20× magnification) and photographed (using INFINITY 1 Lumenera camera and INFINITY ANALYZE and CAPTURE software, v. 6.5.5, Lumenera Corporation, Ottawa, Canada). Visual identification followed the methods and standards presented in [19] regarding microplastics categorization. Visual identification was supported by Fourier transform infrared spectroscopy (FTIR; Cary 630, Agilent/Microscope Spotlight 400, PerkinElmer) to determine the type of plastics recovered. Plastics include the traditional synthetic polymers, while semi-synthetic biobased materials, including modified cellulose, were separately classified. A particle length of >120 μm was set as the lowest identifiable length to correspond to the mesh sizes applied in the method. Therefore, the limit of detection (LOD) is between 5 mm and 120 μm. 

Dietary remains were collected, sterilized in ethanol 70% for 24 h, and stored dry. Prey identification was carried out under a stereoscope microscope. Species and size estimations were carried out using the reference collection stored at the IIM-CSIC in Vigo, the personal reference collection of the second author, and other references available [20,21].

## 3. Results

### 3.1. Identifications of Caves and Seals

A total of eight suitable caves for the species were recorded, mapped, and described along the prospected Albanian coast. Two of the eight caves (caves 1 and 2) were equipped in January 2020 with infrared camera traps as reported in [9] to monitor their effective use by the monk seals (Figure 1b).

Seven active aquaculture sea cage farms were recorded in the area. Those structures are settled in the eastern side of the Karaburun Peninsula, inside the Bay of Vlorë, for farming European sea bass and gilthead sea bream.

On 23 December 2020 (about 1 year after the first installation of the infrared cameras in the caves), a seal was captured (three valid pictures) by the camera in one of the monitored caves (cave 1, Figure 2b). A second capture was recorded on 25 January 2021 from the same cave (nine valid pictures). On 14 February 2021, a seal was “captured” by the cameras (one valid picture) installed in the other cave (cave 2). The limited photographic material recorded by the cameras in these three events did not allow a complete identification of the specimens; however, on the basis of a preliminary evaluation, the morphological characteristics of the animals caught by the cameras depicted subadults. The behaviour of the specimens could not be confirmed due to the limited material recorded.

The thirteen sightings collected through citizen science, documented with pictures (Figure 2c) by RAPA Vlorë from 19 March 2019 until 13 December 2020, analysed and reported in a previous study [9] were also compared with the pictures collected with the camera traps in this study.

The low quality of the pictures from the citizen scientists along with the limited number of pictures from the camera traps did not allow a proper individual identification. Neither was it possible to have a quantitative evaluation of animals frequenting the Albanian coast. However, all pictures depict single animals. The only clear data emerging from the analysis of the pictures were that the animals captured in 2019 appeared younger than the ones captured in 2020 and 2021.

Finally, on 4 March 2022, two individuals were captured by a camera trap in one of the monitored caves. They were not included in this study. However, such data were considered in the discussion.

### 3.2. Identifications of Prey Remains and Microplastics

**Figure 3:**
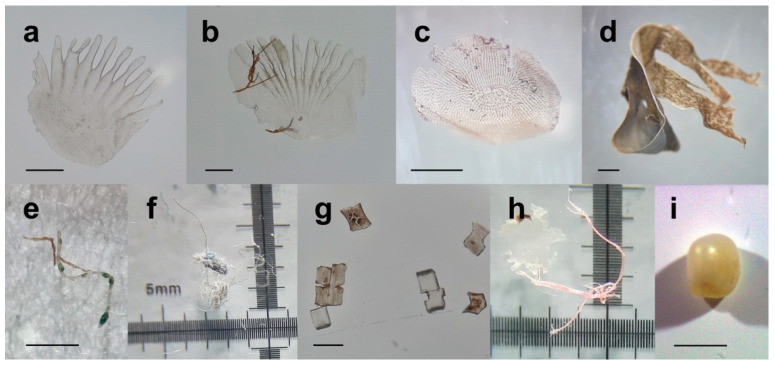
Items identified in Mediterranean monk seal recovered in Albania: (**a**) sea bass scale, (**b**) (possible) sea bream scale, (**c**) garfish scale, (**d**) Posidonia, (**e**) piece of nylon net, (**f**) lump of fibres (cellulose), (**g**) pieces from unknown origin (possibly pieces of fish spines), (**h**) pink fibre (polymer identification not carried out), and (**i**) pearl (scale bar: 1 mm).

The prey identification was carried out on the remains of the scat after washing. Scales were the most identified items. Three possible fish species were identified: a sparid, most probably a gilt-head sea bream (*Sparus aurata*); a sea bass (*Dicentrarchus labrax*); and a garfish (*Belone belone*). The estimated sizes of the fish were 20 cm, 25 cm, and 17 cm, respectively (Table 1 and Figure 3).

Additional information could not be provided due to the high erosion and condition of the scales. It was not possible to distinguish whether the scales belonged to wild or aquaculture sea bream and sea bass, as previously indicated [20]. No other items could be identified, although pieces of seagrass (*Posidonia oceanica*) and a small pearl were also present (Table 1, Figure 3).

A total of 32 publications containing information on the diet of the Mediterranean monk seal, based on the analysis of stomach contents and scats, directly inferred from animal predatory behaviour and stable isotopes, were reviewed (Table 2) [8,10,11,12,13,22,23,24,25,26,27,28,29,30,31,32,33,34,35,36,37,38,39,40,41,42,43,44,45,46,47,48]. The most abundant information (41%) was published in scientific journals, 25% as conference book of abstracts and proceedings, 13% in scientific reports, and the rest in different types of publications (e.g., reports, PhD thesis, newsletters). Most of the information was obtained from stomach contents (72%) and scats (22%). Only two publications investigated several samples [12,13], and only one of them was a scientific publication [12].

The presence of anthropogenic material and microplastics was detected in the sample. Particles >120 µm were isolated from the smallest sieve after alkaline digestion. Procedural blanks (*n* = 3) were carried out to monitor for airborne contamination in the laboratory. A single black fibre (132 µm) was identified in the blanks. It was suspected that the black fibres came from the dark clothing worn under laboratory coats by the laboratory team. No similar particles were found in the scat sample; therefore, no correction was made to the data. 

Anthropogenic materials were identified after the material on the 120 µm sieve was digested with KOH, isolated, and photographed before being run through µ-FTIR. A total of 14 microfibres were classified as anthropogenic materials of which six were confirmed to be plastic polymers (polypropylene × 2, polyethylene terephthalate × 2, polyamide × 2, and acrylic × 1, Table 3, Figure 3). The remaining eight were identified as cellulose, but it was not possible to distinguish between natural and modified cellulose; the bright colour suggested a high possibility that the particles were made of modified cellulose (Table 3). There were also many small “clear” fragments that did not produce clear FTIR spectra. In addition, a piece of nylon, indicative of the nets used in the fishing or aquaculture industry, was identified (Table 3, Figure 3), but the polymer identification with µ-FTIR was not possible.

## 4. Discussion

The habitat use of monk seals in the Albanian coasts was previously reported in general terms, with no specific reference on caves, usually referred over a wide geographical area. Additionally, there has been a limited scientific evaluation (based on effective data) on population numbers, reproductive activities, and/or specific habitat use (as recently reviewed in [9]). This information is relevant to understand the conservation, management, and ecology of an endangered species such as the Mediterranean monk seals.

Seal pictures collected from 2019 to the present might refer to the same individual frequenting the area. This is a precautionary evaluation not to overestimate the actual number. The recovery of a monk seal scat in a marine cave along the Peninsula of Karaburun is the first evidence of specific habitat used by the species in Albania. The seal scat retrieved might reasonably belong to the animals previously encountered and photographed by the citizen science activity the same year. All occasional photographic records of the specimens recorded in 2019–2020 always captured a single individual at a time, with the first ones capturing younger individuals than the ones in the latest [9]. The pictures obtained with the camera traps in the winter of 2020–2021 similarly framed a subadult. However, the data collected did not allow any quantitative or qualitative evaluation of seals frequenting the area, even if it cannot be excluded that the area might be frequented by more than one individual. 

Monk seal sightings were also recorded in the area before this current investigation [4,18,49], but they cannot be ascribed to the same specimens encountered and analysed in this research. Similarly, occasional sightings were recorded from neighbouring countries, such as Montenegro [50] (Archipelagos-environment and development, *Unpublished Data*) and southeast Italy [51] (Archipelagos-ambiente e sviluppo, Italia, *Unpublished Data*), and the Northern Ionian Sea [52] (Archipelagos-environment and development, *Unpublished Data*). Additionally, a thriving monk seal population inhabits the central Ionian Sea, Greece [53,54,55,56].

Such mammals might move towards a wide area along the Adriatic–Ionian Basin, as recent genetic studies seem to prove, e.g., [57,58]. These movements might eventually lead to the establishment of new reproductive groups, especially if appropriate conservation measures are introduced and contribute to support animal survival. However, cross-photo identification of monk seals among countries has not been performed yet.

Confirmation of the use of the coast by more than one individual was recorded in 2022 (RAPA Vlorë, *Unpublished Data*). Two individuals were captured by a camera, one of which appeared to be younger than the other. With these data, it might be too early to assert that the species is reproducing in the area since the birth was not recorded. However, distances between the southern coasts of Albania, Northern Ionian Sea, and Southern Apulia (Italy) are within a range of 50–80 km, which is within the seal movement capability of about 40 km/day [59]. This area might be frequented by a low number of seals but with reproductive activity still taking place. In fact, several sightings including pups and juveniles’ seals were recorded in time (Archipelagos - environment and development, *Unpublished Data*).

In terms of anthropogenic activities, this study only considered the fish farms because this species has been recorded as interacting with these structures in other areas [60,61] (Gema Hernandez-Milian, *pers.comm*). Fish farming in Albania was established in 2000. The most important fish farm areas are located along the eastern side of the Karaburun Peninsula, in the Bay of Vlorë outside the limit of the MNP K-S, and present seven of the 16 fish farms [62,63] operating in the country. They all farm gilthead sea bream and European sea bass. In order to evaluate the risks that the Mediterranean monk seals might face in the area, it is important to consider also the possible threats and/or negative interactions with aquaculture facilities.

Studies on the feeding ecology of pinnipeds are usually carried out by collecting scats from their haul-out places because they exhibit large colonies (e.g., more than seven thousand seals around Irish waters [64]), it is a feasible and non-invasive technique, and the recovery of carcasses is rare. The monk seal population in the Mediterranean Sea has been estimated to be between 350 and 450 seals [1,2,3], mainly concentrated along the coast of Greece and Turkey; in addition, several occasional encounters in other areas of the Mediterranean Sea, including the Adriatic and North Ionian Sea, were recorded, e.g., [9,50,51] (Archipelagos-environment and development, *Unpublished Data*).

Considering the lower population numbers of the Mediterranean monk seal and the difficulties in recovering carcasses, the use of scats represents a practical and efficient tool to the feeding ecology of this study. This might provide a better approach to the diet of the animals (e.g., [14]).

Up to date, the trophic ecology of this species has been mainly inferred from direct observations of the feeding behaviour of the animals or from information retrieved by fishers. Occasional studies were performed, few of which present consistent data, analysing the stomach content of specimens, skin and hairs, bones, scats, and regurgitates, and inferred from the analysis of preys (listed in Table 2). Dietary studies on the Mediterranean monk seal are limited and mainly opportunistic. In fact, despite the amount of publications available, contributing to the study of the feeding ecology of the species, only two presented a considerable number of samples [12,13] and only one of them was a scientific publication. The most comprehensive study used the opportunistic recovery of carcasses from 1997 to 2008 along Greek coastal areas providing information on the species diet through 27 dead seals, with an average of two to three seals per year [12]. In order to investigate the feeding ecology of this species, it is essential to have good knowledge of the caves’ location, habitat use, and identification of prey. This research was based on the identification of prey remains using the traditional methodology to obtain the size and biomass of the prey. However, a bias regarding the high erosion of the prey remains (the stronger among the most common European seals) and intensifies the arduous task of identifying the seals’ prey as previously reported (e.g., [33] Gema Hernandez-Milian, *pers.comm.*). The scarcity of comprehensive studies on this topic points out the relevance to focus on the trophic ecology of this top predator in the Mediterranean Sea and the role within its ecosystems. 

Only a few studies on microplastics incidence aimed to assess possible threats for the species and its presence in monk seal scat following accurate protocols [65,66]. Microdebris was also found in the scat collected in Albania, and the items recovered showed potential trophic transfer (as in previous studies). Although potential airborne contamination coming from the cave cannot be excluded, the low exposure to open air of the cave’s internal chamber allows us to consider its absence. This study reported similar information with other studies on marine mammals [65,66,67] where small dark colour fibres were the most identified microplastics items. The items found in the scat confirmed that microplastics are transferred within the food webs, and it is important to investigate the impacts that these pollutants might cause to top predators in the Mediterranean Sea.

## 5. Conclusions

Following the Marine Strategy Framework Directive (MSFD) established by the European Parliament in 2008 (Directive 2008/56/EC), the good environmental status (GES) of marine ecosystems should be assessed through 11 quality descriptors. These descriptors included some aspects of top predators such as abundance and distribution (D1, biodiversity), trophic information (D4, food webs), and impact of marine debris (D10, marine litter). The present study confirmed the relevance of the Albanian coast for the Mediterranean monk seal, which might represent an important ecological corridor for the species recovery along the Adriatic–Ionian region [9,68]; therefore, information obtained from this study could be included in D1. In order to enhance this information, additional long-term monitoring and conservation activities are recommended in order to increase the local knowledge of the species presence and frequentation of the coast and to extend protection actions over a network of countries within a wide regional approach. In addition, the collection of samples using non-invasive techniques demonstrated that the acquired information is of high value to understand the trophic ecology of the species, investigate the trophic transfer of anthropogenic debris, and provide information to assess the GES in the study area. Additional research should be performed in the future, such as collecting samples for genetic analysis in combination with the traditional methodology for a better description of prey ingested by seals.

## Figures and Tables

**Table 1:** Prey identified and anthropogenic debris incidence in monk seal scat recovered in Albania.

Remains Identified	Length (mm)	Item Identified
Beloniformes		
*Belone belone*	17	Scale and bone
Perciformes		
*Dicentrarchus labrax*	25	Scale
Sparidae *	20	Scale
Vegetation		
*Posidonia oceanica*	13	Pieces of Posidonia
Anthropogenic debris	>120 µm	Nylon (net), fibres, and unknown fragments

* Likely *Sparus aurata*.

**Table 2:** Review of Mediterranean monk seal feeding ecology studies.

Type of Analysis	n° of Samples/Specimens	Type of Contribution	Reference
Stomach	1 stomach	Scientific publication	Carruccio 1893 [22]
Stomach	2 stomachs	Scientific publication	Bacescu 1948 [23]
Stomach	1 stomach	Scientific publication	Salnikov 1959 [24]
Stomach	1 stomach	Scientific publication	Sergeant et al., 1978 * [25]
Stomach	1 stomach	Scientific report	Melo Machado 1979 * [26]
Stomach	3 stomachs	Proceedings	Soriguer 1979 [27]
Stomach, scat	2 stomachs, unknown scats	Scientific report	Marchessaux 1989 ‡ [28]
Stomach, scat	4 stomachs, 10 scats	PhD thesis	Marchessaux 1989 ‡ [10]
Stomach	1 stomach	Scientific report	Cebrian et al., 1990 # [29]
Stomach	3 stomachs	Book	Lopez-Jurado et al., 1995 [30]
Stomach, regurgitate	Unknown	Conference contribution	Boutiba and Abdelghani 1996 [31]
Inferred from prey	8 Preys	Proceedings	Margaritoulis et al., 1996 [32]
Stomach, scat, regurgitate	1 stomach, unknown scat, and regurgitates	PhD thesis	Cebrian 1998 # [33]
Scat	1 scat	Scientific publication	van Bree and Panou 2000 [34]
Stomach	2 stomachs	Scientific publication	Salman et al., 2001 [11]
Stomach, scat	9 stomachs, 5 scat	Book	Gonzalez et al., 2006 [8]
Stable isotopes (skin)	44 specimens	Conference contribution	Karamanlidis et al., 2009 [35]
Stable isotopes (bone)	12 specimens	Scientific publication	Pinela et al., 2010 [36]
Stomach	1 stomach	Scientific publication	Karamanlidis et al., 2011 [37]
Inferred from prey	21 preys	Newsletter	Margaritoulis and Touliatou 2011 [38]
Stomach, scat	27 stomachs, 14 scats	Scientific publication	Pierce et al., 2011 ¥ [12]
Stomach	18 stomachs	Conference contribution	Muñoz-Cañas et al., 2012 [39]
Stable isotopes (hair)	23 specimens	Scientific publication	Karamanlidis et al., 2014 [40]
Scat	8 scats	Conference contribution	Bundone et al., 2015 [41]
Stomach	1 stomach	Conference contribution	Toney et al., 2015 # [42]
Stomach	1 stomach	Scientific publication	Toney et al., 2016 # [43]
Stomach	35 stomachs	Conference contribution	Hernandez-Milian et al., 2018 [13]
Inferred from prey	13 preys	Conference contribution	Kapiris et al., 2018 [44]
Stomach	1 stomach	Scientific publication	Kirac and Ok 2019 [45]
Stomach	2 stomachs	Conference contribution	Pietroluongo et al., 2019 [46]
Stomach	1 stomach	Scientific report	Pires et al., 2020 [47]
Inferred from prey	23 prey/turtles	Scientific publication	Snape et al., 2022 [48]

* Refers to the same unpublished data of 1957 (G.E. Maul); ‡ The latter might include the data of the former; # The latter include the observation of the former; ¥ Absence of identifiable hard prey remains from scats.

**Table 3:** Anthropogenic debris identified using µ-FTIR. N.id.: it was not possible to identify because they were too small or they were lost when sent for laboratory analysis.

Shape	Colour	Polymer	Size (µm)
Bead	White	N.id.	900
Fibre	Blue	Acrylic	571
Fibre	Blue	Cellulose	1058
Fibre	Blue	Cellulose	338
Fibre	Blue	Cellulose	1808
Fibre	Red	Cellulose	3915
Fibre	Blue	Polypropylene	270
Fibre	Black	Cellulose	1305
Fibre	Black	Cellulose	16,778
Fibre	Red	Cellulose	675 (Figure 3h)
Fibre	Black	Polyethylene terephthalate	2565
Fibre	White	Polypropylene	>15 (Figure 3f)
Fibre	Brown	Nylon	10,983
Fibre	White	Polyethylene terephthalate	11,111
Fibre	Blue	Cellulose	1035
Fibre	White and green	Nylon	5700 (Figure 3e)
Fragments *	Clear and white	N.id.	N.id.

* 9 fragments were visually identified.

## Data Availability

None of the data was deposited in an official repository. All data and pictures included in the manuscript and related to a specific location of the habitat use of the Mediterranean monk seals cannot be shared in accordance with the confidentiality related to conservation issues. Data are available upon request.
